# Mitochondrial DNA copy number is associated with risk of head and neck squamous cell carcinoma in Chinese population

**DOI:** 10.1002/cam4.1452

**Published:** 2018-04-19

**Authors:** Lihua Wang, Hong Lv, Pei Ji, Xun Zhu, Hua Yuan, Guangfu Jin, Juncheng Dai, Zhibin Hu, Yuxiong Su, Hongxia Ma

**Affiliations:** ^1^ Department of Epidemiology School of Public Health Nanjing Medical University Nanjing 211166 China; ^2^ Jiangsu Key Laboratory of Oral Diseases Nanjing Medical University Nanjing 210029 China; ^3^ Jiangsu Key Lab of Cancer Biomarkers, Prevention and Treatment Collaborative Innovation Center of Cancer Medicine Nanjing Medical University Nanjing 211166 China; ^4^ Oral and Maxillofacial Surgery Faculty of Dentistry The University of Hong Kong Hong Kong

**Keywords:** Association analysis, case–control study, head and neck, mitochondrial DNA copy number, squamous cell carcinoma

## Abstract

Mitochondria show the special role in cellular bioenergy and many essential physiological activities. Previous researches have suggested that variations of mitochondrial DNA copy number contribute to development of different types of carcinomas. However, the relationship of mtDNA copy number in peripheral blood leukocytes (PBLs) with the risk of head and neck squamous cell carcinoma (HNSCC) is still inconclusive. We investigated the association of mtDNA with HNSCC risk through a case–control study including 570 HNSCC cases and 597 cancer‐free controls. mtDNA copy number in PBLs was measured by real‐time qPCR. Logistic regression was performed to estimate the association between the mtDNA copy number in PBLs and HNSCC risk. A U‐shaped relation between the mtDNA copy number and HNSCC risk was found. Compared with those in the second quartile group, the adjusted odds ratios (ORs) and 95% confidence interval (CI) for those in the first and the forth quartile groups were 1.95 (1.37–2.76) and 2.16 (1.53–3.04), respectively. Using restricted cubic spline analysis, we confirmed such a significant U‐shaped relation. Furthermore, the U‐shaped association remained significant in different subgroups stratified by age, gender, tobacco smoking, and alcohol consumption. Both extremely low and high mtDNA copy numbers had significant associations with the increased HNSCC risk.

## Background

Mitochondria are complex and dynamic organelle 1 containing 2–10 mitochondrial DNA (mtDNA) molecules 2, which explore a significant role in the function and longevity of the vast majority of eukaryotic cell and organisms 1. Human mitochondrial DNA is a circular double‐stranded DNA molecule of 16.6 kb, and encodes 33 genes responsible for its protein synthesis (22 tRNAs and 2 rRNAs) and the respiratory chain (13 polypeptides) 2, 3. Compared to nuclear genome, the lack of introns and protective histones makes mtDNA susceptible to damage from reactive oxygen species (ROS) and other damaging agents 4. Additionally, alterations of mtDNA via inactivating genetic mutations or depleting mtDNA copy number may induce the damage of mitochondrial respiration and contribute to multiple disease, such as encephalopathies, neuropathies, and cancers 5, 6, 7.

The mtDNA copy number varies greatly between different tissues 8, but remains relatively stable in normal cells 9. Researchers have demonstrated that abnormal mtDNA copy number is involved in the development of various cancers, such as lung cancer 10, papillary thyroid carcinomas 11, breast cancer 12, hepatic cell carcinomas 13, gastric cancers 14, and ovary cancer 15. Furthermore, epidemiological researches have explored the significance of the mtDNA copy number in PBLs, which can serve as a potential biomarker of cancer risk prediction. However, the results were inconclusive in different cancers. For example, higher mtDNA copy number was related to the elevated risk of renal cell carcinoma 16, but lower mtDNA copy number increased the risk of endometrial cancer and melanoma 17, 18.

Head and neck cancer mainly includes squamous cell carcinomas situating in oral cavity, oropharynx, hypopharynx, and larynx 19. It ranks sixth among all cancers and accounts for about 4% new cases and 5% deaths of all malignancies around the world 20. In China, the incidence of HNSCC is relatively low; however, the total number of cases is large because of its vast population. It was reported that there were about 74.5 thousand new cases and 36.6 thousand cases died of HNSCC in 2015 21. Some reports and studies have focused on the role of mtDNA copy number in HNSCC development 22, 23, 24, 25, 26, 27; however, the sample sizes were relatively small (total number of cases and controls: 155–500) and their results were inconsistent 22, 23, 24, 25, 26, 27. Thus, we examined the relationship between mtDNA copy number and HNSCC risk in a relatively larger case–control study (570 HNSCC cases and 597 controls) in China.

## Methods

### Study participants

A total of 570 newly diagnosed HNSCC cases were recruited through Jiangsu Stomatological Hospital and the First Affiliated Hospital of Nanjing Medical University, since January 2009 to May 2013. All cases were first diagnosed with HNSCC cancer in the hospitals and did not receive any cancer‐related treatment (chemotherapy/radiotherapy) before this study. A total of 597 cancer‐free controls were randomly chosen from more than 30,000 individuals who participated in a screening program for noninfectious diseases in Jiangsu province, China. Cases and controls were frequency‐matched on age and sex. After signed the informed consent, all participants completed the standard questionnaire through the face‐to‐face interview. Data were collected on demographics including age and gender, and some exposure factors such as tobacco smoking and alcohol consumption. Individuals who had smoked an average of one or more cigarettes per day for at least 1 year were defined as smokers, and those who had more than one alcoholic drink per day were considered as drinkers. A fasting blood sample (~5 mL) was collected in the morning when each patient was admitted to hospital and had not received any treatment. Additionally, the fasting blood sample was also taken from each control for DNA extraction. All subjects were genetically unrelated Han Chinese. The study was approved by the Ethics Committee of Nanjing Medical University.

### Measurement of mtDNA copy number

DNA was extracted from PBLs by traditional phenol–chloroform method. A quantitative reverse transcription PCR‐based method was used to determine the relative mtDNA copy number in PBLs according to procedures described previously 28, 29. Briefly, two primer sets were respectively designed to amplify the ND1 gene in mtDNA and the nuclear gene HGB to measure the mtDNA copy number as described by Liu et al. 28. The mitochondrial gene quantitative PCR (qPCR) primers were ND1‐F (5′‐CCCTAAAACCCGCCACATCT‐3′) and ND1‐R (5′‐GAGCGATGGTGAGAGCTAAGGT‐3′), while the nuclear gene (HGB) qPCR primers were HGB‐1 (5′‐GAAGAGCCAAGGACAGGTAC‐3′) and HGB‐2 (5′‐CAACTTCATCCACGTTCACC‐3′). The final primer concentration used for the real‐time PCR assay was 10 μmol/μL. The mtDNA copy number was expressed as the ratio of *ND1* and *HGB* threshold cycle numbers in individual samples, and it was calculated in accordance with the formula 28: mtDNA copy number = 2 × 2^(Ct(HGB)‐Ct(ND1))^. A genomic DNA pool from five healthy donors was serially diluted 1:2 to generate a standard curve ranging from 0.625 to 20 ng of DNA for which the *R* ^2^ for each standard curve was 0.99 or greater. Samples that fell outside the range defined by the standard curves were rerun at a different concentration to ensure that they were amplified within the linear range. The PCR mixture in each well included 1× SYBR Green PCR Master Mix (Applied Biosystems, Foster City, CA, USA), ND1‐R (or HGB‐1) primer, ND1‐F (or HGB‐2) primer and genomic DNA. All samples were evaluated by ABI PRISM 7900HT Sequence Detection System in duplicate on a 384‐well plate. Especially, the PCRs for mtDNA and HGB of the same samples were carried out on separate 384‐well plates in the same well positions. Quality control samples were run with the case–control sets in each batch. Mean values within and between batch coefficients of variation based on the quality control samples were 1.86% and 6.16%, respectively. The coefficient of variation is an effect size measure, which is defined as the ratio of the standard deviation to the mean. All laboratory personnel were blinded to the case or control status.

### Statistical analysis

Pearson's chi‐square test was used to evaluate the differences in demographics (age, sex) and risk factors (tobacco smoking, alcohol consumption) between cases and controls. Additionally, rank‐sum test was conducted to detect mtDNA copy number differences in PBLs between cases and controls, and between subgroups with different age, sex, smoking, and drinking. We categorized mtDNA copy number into three or four groups based on its tertile or quartile distributions in controls, respectively, aiming to detect the association between mtDNA copy number and the risk of HNSCC. Unconditional logistic regression was used to compute ORs and 95% CI in each mtDNA copy number quartile, adjusting for age, sex, smoking and drinking status. Restricted cubic spline analysis in logistic regression model was used to derive the shape of relationship between mtDNA copy number and HNSCC risk. All tests for *P* values were two‐sided, with *P *<* *0.05 considered statistically significant. Stata/SE 11.0 (StataCorp LP, Lakeway Drive College Station, Texas, USA) and SAS version 9.1.3 (SAS Institute, NC, USA) were used for statistical analyses.

## Result

### Primary information

Table 1 shows the characteristics of subjects in our study. A total of 570 HNSCC cases and 597 controls were included in the analysis. Differences of age and sex were nonsignificant (*P *>* *0.05), and the mean age and standard deviation (SD) for cases and controls were 61.07 ± 10.88 and 59.62 ± 9.35, respectively. However, HNSCC cases (44.21%) had higher proportion of drinkers than that in the cancer‐free controls (32.16%) (*P *<* *0.05), while that of smokers between two groups were similar (*P *>* *0.05). More than 80% of cases were those with cancer of oral cavity, nearly 20% with larynx and around 2% with oropharynx and others.

**Table 1:** Distributions of characteristics in HNSCC cases and controls

Variables	Cases *N* (%)	Controls *N* (%)	*P* a
All subjects	570 (100.00)	597 (100.00)	
Age			
<60	277 (48.60)	285 (47.74)	0.769
≥60	293 (51.40)	312 (52.26)	
Gender			
Females	213 (37.37)	223 (37.35)	0.996
Males	357 (62.63)	374 (62.65)	
Smoking status			
Never	312 (54.74)	344 (57.62)	0.321
Ever	258 (45.26)	253 (42.38)	
Drinking status			
Never	318 (55.79)	405 (67.84)	**<0.001**
Ever	252 (44.21)	192 (32.16)	
Tumor sites			
Oral cavity	458 (80.53)		
Oropharynx	9 (1.58)		
Larynx	100 (17.54)		
Other^b^	3 (0.53)		

HNSCC, head and neck squamous cell carcinoma.

a Two‐sided chi‐square test.

b Including nasal sinuses, parotid, and salivary gland.

P value in bold is statistically significant.

We also evaluated mtDNA copy number among cancer‐free controls with various characteristics, and no apparent difference was found between subgroups by age, gender, tobacco smoking or alcohol drinking (Table 2).

**Table 2:** Distributions of mtDNA copy number among cancer‐free controls

Selected variables	*N*	mtDNA copy number median (quartile)	*P* a
Overall	597	3.50 (1.93–11.22)	
Age			
<60	285	3.33 (1.94–9.23)	0.273
≥60	312	3.90 (1.89–12.84)	
Gender			
Male	374	3.46 (1.83–11.21)	0.48
Female	223	3.59 (2.06–11.85)	
Smoking status			
Never	344	3.51 (2.00–11.83)	0.627
Ever	253	3.47 (1.76–10.88)	
Drinking status			
Never	405	3.59 (1.90–12.09)	0.684
Ever	192	3.43 (2.00–9.44)	

a Derived from rank‐sum test for mtDNA copy number between subgroups divided by characteristics.

### The association of mtDNA copy number with HNSCC risk

**Figure 1:**
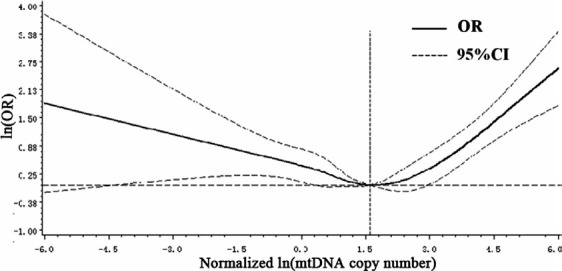
Association between mtDNA copy number and HNSCC risk based on restricted cubic spline function in the logistic regression model.

The mtDNA copy number significantly elevated in HNSCC cases than that in controls (median: 4.33 vs. 3.50, *P *=* *0.016). Then, we categorized participants into four groups based on quartile distribution of mtDNA copy number in controls and evaluated the relationship between mtDNA copy number and HNSCC risk. Compared with the subjects in the second quartile group, a significantly increased risk of HNSCC was detected in both groups with the lowest quartile and highest quartile groups of relative mtDNA copy number [adjusted OR (95% CIs), 1.95 (1.37–2.76), 2.16 (1.53–3.04)] (Table 3). We also categorized participants into three groups based on tertile distribution of mtDNA copy number in controls and the result confirmed the finding from quartile groups (Table 3). Further, we also found a significant U‐shaped association using the restricted cubic spline analysis (Fig. 1).

**Table 3:** Association between mtDNA copy number (mtDNAcn) and HNSCC risk

Groups of mtDNA copy number by control category		Controls *N* (%)	Cases		
			*N* (%)	Adjusted OR (95% CI) a	*P* a
Quartile	<1.93	150 (25.13)	165 (28.95)	**1.95 (1.37–2.76)**	**<0.001**
	1.93–3.50	149 (24.96)	89 (15.61)	1.00 (reference)	—
	3.50–11.22	149 (24.96)	119 (20.88)	1.34 (0.93–1.92)	0.116
	>11.22	149 (24.96)	197 (34.56)	**2.16 (1.53–3.04)**	**<0.001**
Tertile	<2.38	199 (33.33)	195 (34.21)	**1.62 (1.19–2.19)**	**0.002**
	2.38–6.49	199 (33.33)	128 (22.46)	1.00 (reference)	—
	≥6.49	199 (33.33)	247 (43.33)	**1.90 (1.41–2.55)**	**<0.001**

a Derived from logistic regression with an adjustment for age at blood collection, sex, smoking and drinking status.

P value in bold is statistically significant.

When the above association was stratified by age, gender, tobacco smoking and alcohol consumption, the U‐shaped association still existed in both young and old subjects, women and men, nonsmokers and smokers, nondrinkers and drinkers and no significant heterogeneity was observed in every two strata (*P *>* *0.05). (Table 4).

**Table 4:** Stratified analyses of association between mtDNA copy number and HNSCC

Variables		mtDNA copy number in quartile			
		1st group	2nd group	3rd group	4th group
Age					
<60	Cases/Controls	82/69	46/81	62/72	87/63
	Adjusted OR (95% CI)^a^	2.17 (1.33–3.54)	1.00	1.51 (0.91–2.50)	2.36 (1.44–3.87)
≥60	Cases/Controls	83/80	43/69	57/77	110/86
	Adjusted OR (95% CI)^a^	1.78 (1.08–2.92)	1.00	1.20 (0.72–2.02)	2.09 (1.29–3.38)
Gender					
Male	Cases/Controls	110/101	57/91	75/89	115/93
	Adjusted OR (95% CI)	1.88 (1.22–2.92)	1.00	1.36 (0.86–2.15)	1.99 (1.28–3.09)
Female	Cases/Controls	55/48	32/59	44/60	82/56
	Adjusted OR (95% CI)^a^	2.10 (1.17–3.78)	1.00	1.25 (0.69–2.26)	2.54 (1.46–4.44)
Smoking					
No	Cases/Controls	93/78	50/93	67/85	102/88
	Adjusted OR (95% CI)^a^	2.16 (1.36–3.41)	1.00	1.46 (0.91–2.34)	2.08 (1.33–3.27)
Yes	Cases/Controls	72/71	39/57	52/64	95/61
	Adjusted OR (95% CI)^a^	1.69 (0.97–2.95)	1.00	1.14 (0.64–2.03)	2.36 (1.36–4.09)
Drinking					
No	Cases/Controls	100/103	55/94	63/102	100/106
	Adjusted OR (95% CI)^a^	1.73 (1.11–2.68)	1.00	1.06 (0.67–1.69)	1.58 (1.02–2.45)
Yes	Cases/Controls	65/46	34/56	56/47	97/43
	Adjusted OR (95% CI)^a^	2.27 (1.27–4.05)	1.00	1.97 (1.10–3.54)	3.60 (2.05–6.34)
Oral cancer					
No	Cases/Controls	39/149	18/150	29/149	26/149
	Adjusted OR (95% CI)^a^	2.17 (1.16–4.06)	1.00	1.70 (0.89–3.27)	1.46 (0.75–2.83)
Yes	Cases/Controls	126/149	71/150	90/149	171/149
	Adjusted OR (95% CI)^a^	1.91 (1.31–2.78)	1.00	1.27 (0.86–1.88)	2.39 (1.66–3.44)

a Derived from logistic regression with an adjustment for age at blood collection, sex, smoking and drinking status.

## Discussion

In the present study, we conducted a case–control study to evaluate the relationship between mtDNA copy number and HNSCC risk and found that both extremely low and high mtDNA copy number had significant associations with the increased risk of HNSCC. These findings suggested that there was a U‐shaped association between mtDNA copy number and HNSCC risk, providing a novel perspective for understanding the significance of mtDNA in the etiology of HNSCC.

Mitochondria play critical roles in the maintenance of functionally competent organelles, and dysfunction of mitochondrial has been involved in various human pathologies including human cancers 6, 30. Abnormal mtDNA copy number may confer cancer development depending on a variety of mechanisms, including increased production of reactive oxygen species, decreased oxidative phosphorylation, increased expression of prosurvival proteins, and resistance to apoptosis 29, 31, 32, 33, 34, 35, 36, 37. For example, the lack of mtDNA altered gene expression in mitochondrial and caused an enhanced production of reactive oxygen species and deficiency in oxidative phosphorylation, inducing disrupted function of cells 38. Furthermore, multiple epidemiologic studies explored its significance as a marker in PBLs for tumor risk and found low mtDNA copy number would increase the risk of several cancers, such as breast tumors 12, 31, hepatic cell carcinomas 13 and esophageal adenocarcinoma 39. However, some other studies indicated inverse relation between mtDNA copy number and risk of some special carcinomas including renal cell carcinoma 16, lung cancer 10, 40 and papillary thyroid carcinoma 11. The biologic mechanism for high mtDNA copy number and the increased risk of cancer is still not fully understood. We speculated that increased mtDNA copy number in PBLs may be markers of elevated oxidative stress and ROS‐mediated DNA damage, and thus, higher mtDNA copy number may compensate for mtDNA damage/dysfunction 34, 35, 37. In the meantime, ROS produced by increased mitochondria may lead to more oxidative damage to some intracellular constituents, and then contribute to the initiation and promotion of carcinogenesis 41.

So far, six studies have discussed the association between mtDNA copy number and head and neck cancer (HNC) risk 22, 23, 24, 25, 26, 42, but results were inconsistent. For example, Lin et al. reported that the mtDNA copy number was significantly higher in HNC patients 22; however, the study by Takeda et al. reported that mtDNA copy number was decreased in tumor compared to that in normal tissue 26. Interestingly, we first found a U‐shaped relationship between mtDNA copy number and HNSCC risk, which was similarly reported by a nested case–control study that showed the lowest and highest quartile of mtDNA copy number were significantly associated with the increased risk of colorectal cancer 34. Thus, real associations between mtDNA copy number and risk of some cancers might be not a simple linear relationship. Previous studies on HNSCC failed to found such association, possibly because of relatively small sample size or lack of more detailed analysis. Actually, the U‐shaped association found in our study was biologically possible. Both extremely low and high copy number of mtDNA may lead to cell dysfunction and abnormal cell microenvironment, and finally result in the initiation and promotion of cancer 28, 29, 31, 32, 34, 35, 37, 43. Further studies with larger sample size and more rigorous design are warranted to confirm these finding.

Some limitations in the present study need to be pointed out. First of all, the statistical power may be limited because of a relatively modest sample size in this study, especially for stratification analysis. Secondly, this study was a case–control design, and we could not completely rule out the possibility of “reverse causal association.” However, we have only included newly diagnosed and previously untreated patients to reduce the influence of disease status and treatment on mtDNA copy number. Thirdly, the mtDNA copy was measured in PBLs, but not in target tissues. Thus, it is unclear whether this change is really related to the etiology of head and neck cancer. Fourthly, mtDNA copy number was examined only once and repeated measurements over time were unavailable. Finally, this study was only conducted in Chinese Han population, so the external validity of conclusions might be limited.

In summary, this is the first study to show that both extremely high and low mtDNA copy numbers were related to HNSCC risk. The findings provided valuable clues for better understanding the role of mtDNA copy number in the development of HNSCC. However, prospective designed studies with a sufficient sample size are needed to verify our findings.

## Declarations

*Ethics approval and consent to participate*: This study was approved by the Ethics Committee of Nanjing Medical University.

*Availability of data and materials*: The data sets used and/or analyzed during the current study are available from the corresponding author on reasonable request.

## Conflict of Interest

The authors declare no conflict of interests.

## References

[1] Shadel, G. S. 2008 Expression and maintenance of mitochondrial DNA: new insights into human disease pathology. Am. J. Pathol. 172:1445–1456. 18458094 10.2353/ajpath.2008.071163 PMC2408405

[2] Xing, J. , M. Chen , C. G. Wood , J. Lin , M. R. Spitz , J. Ma , et al. 2008 Mitochondrial DNA content: its genetic heritability and association with renal cell carcinoma. J. Natl Cancer Inst. 100:1104–1112. 18664653 10.1093/jnci/djn213 PMC2720693

[3] Chatterjee, A. , S. Dasgupta , and D. Sidransky . 2011 Mitochondrial subversion in cancer. Cancer Prev. Res. (Phila.) 4:638–654. 21543342 10.1158/1940-6207.CAPR-10-0326 PMC3298745

[4] Pinz, K. G. , and D. F. Bogenhagen . 1998 Efficient repair of abasic sites in DNA by mitochondrial enzymes. Mol. Cell. Biol. 18:1257–1265. 9488440 10.1128/mcb.18.3.1257 PMC108838

[5] El‐Hattab, A. W. , and F. Scaglia . 2013 Mitochondrial DNA depletion syndromes: review and updates of genetic basis, manifestations, and therapeutic options. Neurotherapeutics 10:186–198. 23385875 10.1007/s13311-013-0177-6 PMC3625391

[6] Wallace, D. C. 2012 Mitochondria and cancer. Nat. Rev. Cancer 12:685–698. 23001348 10.1038/nrc3365 PMC4371788

[7] Ju, Y. S. , L. B. Alexandrov , M. Gerstung , I. Martincorena , S. Nik‐Zainal , M. Ramakrishna , et al. 2014 Origins and functional consequences of somatic mitochondrial DNA mutations in human cancer. Elife 3:e02935. doi: 10.7554/eLife.02935. 10.7554/eLife.02935 PMC4371858 25271376

[8] Lan, Q. , U. Lim , C. S. Liu , S. J. Weinstein , S. Chanock , M. R. Bonner , et al. 2008 A prospective study of mitochondrial DNA copy number and risk of non‐Hodgkin lymphoma. Blood 112:4247–4249. 18711000 10.1182/blood-2008-05-157974 PMC2582005

[9] Clay Montier, L. L. , J. J. Deng , and Y. Bai . 2009 Number matters: control of mammalian mitochondrial DNA copy number. J. Genet. Genomics 36:125–131. 19302968 10.1016/S1673-8527(08)60099-5 PMC4706993

[10] Bonner, M. R. , M. Shen , C. S. Liu , M. Divita , X. He , and Q. Lan . 2009 Mitochondrial DNA content and lung cancer risk in Xuan Wei, China. Lung Cancer 63:331–334. 18691788 10.1016/j.lungcan.2008.06.012 PMC2966769

[11] Wang, Y. , V. W. Liu , W. C. Xue , P. C. Tsang , A. N. Cheung , and H. Y. Ngan . 2005 The increase of mitochondrial DNA content in endometrial adenocarcinoma cells: a quantitative study using laser‐captured microdissected tissues. Gynecol. Oncol. 98:104–110. 15921730 10.1016/j.ygyno.2005.04.015

[12] Mambo, E. , A. Chatterjee , M. Xing , G. Tallini , B. R. Haugen , S. C. Yeung , et al. 2005 Tumor‐specific changes in mtDNA content in human cancer. Int. J. Cancer 116:920–924. 15856456 10.1002/ijc.21110

[13] Lee, H. C. , S. H. Li , J. C. Lin , C. C. Wu , D. C. Yeh , and Y. H. Wei . 2004 Somatic mutations in the D‐loop and decrease in the copy number of mitochondrial DNA in human hepatocellular carcinoma. Mutat. Res. 547:71–78. 15013701 10.1016/j.mrfmmm.2003.12.011

[14] Wu, C. W. , P. H. Yin , W. Y. Hung , A. F. Li , S. H. Li , C. W. Chi , et al. 2005 Mitochondrial DNA mutations and mitochondrial DNA depletion in gastric cancer. Genes Chromosom. Cancer 44:19–28. 15892105 10.1002/gcc.20213

[15] Wang, Y. , V. W. Liu , P. C. Tsang , P. M. Chiu , A. N. Cheung , U. S. Khoo , et al. 2006 Microsatellite instability in mitochondrial genome of common female cancers. Int. J. Gynecol. Cancer 16(Suppl 1):259–266. 16515601 10.1111/j.1525-1438.2006.00412.x

[16] Hofmann, J. N. , H. D. Hosgood, 3rd , C. S. Liu , W. H. Chow , B. Shuch , W. L. Cheng , et al. 2014 A nested case‐control study of leukocyte mitochondrial DNA copy number and renal cell carcinoma in the Prostate, Lung, Colorectal and Ovarian Cancer Screening Trial. Carcinogenesis 35:1028–1031. 24398668 10.1093/carcin/bgt495 PMC4004202

[17] Sun, Y. , L. Zhang , S. S. Ho , X. Wu , and J. Gu . 2016 Lower mitochondrial DNA copy number in peripheral blood leukocytes increases the risk of endometrial cancer. Mol. Carcinog. 55:1111–1117. 26258624 10.1002/mc.22373

[18] Meng, S. , I. De Vivo , L. Liang , E. Giovannucci , J. Y. Tang , and J. Han . 2016 Pre‐diagnostic leukocyte mitochondrial DNA copy number and skin cancer risk. Carcinogenesis 37:897–903. 27381830 10.1093/carcin/bgw072 PMC5008249

[19] Lajer, C. B. , E. Garnaes , L. Friis‐Hansen , B. Norrild , M. H. Therkildsen , M. Glud , et al. 2012 The role of miRNAs in human papilloma virus (HPV)‐associated cancers: bridging between HPV‐related head and neck cancer and cervical cancer. Br. J. Cancer 106:1526–1534. 22472886 10.1038/bjc.2012.109 PMC3341860

[20] Suh, Y. , I. Amelio , Urbano T. Guerrero , and M. Tavassoli . 2014 Clinical update on cancer: molecular oncology of head and neck cancer. Cell Death Dis. 5:e1018. 24457962 10.1038/cddis.2013.548 PMC4040714

[21] Chen, W. , R. Zheng , P. D. Baade , S. Zhang , H. Zeng , F. Bray , et al. 2016 Cancer statistics in China, 2015. CA Cancer J. Clin. 66:115–132. 26808342 10.3322/caac.21338

[22] Cheau‐Feng, Lin F. , Y. C. Jeng , T. Y. Huang , C. S. Chi , M. C. Chou , and Tsai S. Chin‐Shaw . 2014 Mitochondrial DNA copy number is associated with diagnosis and prognosis of head and neck cancer. Biomarkers 19:269–274. 24773072 10.3109/1354750X.2014.902101

[23] Dang, S. , Y. Qu , J. Wei , Y. Shao , Q. Yang , M. Ji , et al. 2014 Low copy number of mitochondrial DNA (mtDNA) predicts worse prognosis in early‐stage laryngeal cancer patients. Diagn. Pathol. 9:28. 24499477 10.1186/1746-1596-9-28 PMC3916805

[24] Guo, W. , D. Yang , H. Xu , Y. Zhang , J. Huang , Z. Yang , et al. 2013 Mutations in the D‐loop region and increased copy number of mitochondrial DNA in human laryngeal squamous cell carcinoma. Mol. Biol. Rep. 40:13–20. 23114912 10.1007/s11033-012-1939-7

[25] Mondal, R. , S. K. Ghosh , J. H. Choudhury , A. Seram , K. Sinha , M. Hussain , et al. 2013 Mitochondrial DNA copy number and risk of oral cancer: a report from Northeast India. PLoS ONE 8:e57771. 23469236 10.1371/journal.pone.0057771 PMC3587625

[26] He, Y. , Y. Gong , J. Gu , J. J. Lee , S. M. Lippman , and X. Wu . 2014 Increased leukocyte mitochondrial DNA copy number is associated with oral premalignant lesions: an epidemiology study. Carcinogenesis 35:1760–1764. 24743515 10.1093/carcin/bgu093 PMC4123647

[27] Takeda, D. , T. Hasegawa , T. Ueha , A. Sakakibara , T. Kawamoto , T. Minamikawa , et al. 2016 Decreased mitochondrial copy numbers in oral squamous cell carcinoma. Head Neck 38:1170–1175. 27079936 10.1002/hed.24194

[28] Liu, C. S. , W. L. Cheng , C. F. Lee , Y. S. Ma , C. Y. Lin , C. C. Huang , et al. 2006 Alteration in the copy number of mitochondrial DNA in leukocytes of patients with mitochondrial encephalomyopathies. Acta Neurol. Scand. 113:334–341. 16629770 10.1111/j.1600-0404.2006.00586.x

[29] Hosnijeh, F. S. , Q. Lan , N. Rothman , Liu C. San , W. L. Cheng , A. Nieters , et al. 2014 Mitochondrial DNA copy number and future risk of B‐cell lymphoma in a nested case‐control study in the prospective EPIC cohort. Blood 124:530–535. 24899624 10.1182/blood-2013-10-532085 PMC4110659

[30] Schon, E. A. , S. DiMauro , and M. Hirano . 2012 Human mitochondrial DNA: roles of inherited and somatic mutations. Nat. Rev. Genet. 13:878–890. 23154810 10.1038/nrg3275 PMC3959762

[31] Yu, M. , Y. Zhou , Y. Shi , L. Ning , Y. Yang , X. Wei , et al. 2007 Reduced mitochondrial DNA copy number is correlated with tumor progression and prognosis in Chinese breast cancer patients. IUBMB Life 59:450–457. 17654121 10.1080/15216540701509955

[32] Liu, J. , and Z. Wang . 2015 Increased oxidative stress as a selective anticancer therapy. Oxid. Med. Cell Longev. 2015:294303. 26273420 10.1155/2015/294303 PMC4529973

[33] Liu, C. S. , C. S. Tsai , C. L. Kuo , H. W. Chen , C. K. Lii , Y. S. Ma , et al. 2003 Oxidative stress‐related alteration of the copy number of mitochondrial DNA in human leukocytes. Free Radic. Res. 37:1307–1317. 14753755 10.1080/10715760310001621342

[34] Thyagarajan, B. , R. Wang , H. Barcelo , W. P. Koh , and J. M. Yuan . 2012 Mitochondrial copy number is associated with colorectal cancer risk. Cancer Epidemiol. Biomarkers Prev. 21:1574–1581. 22787200 10.1158/1055-9965.EPI-12-0138-T PMC3437007

[35] Lee, H. C. , and Y. H. Wei . 2005 Mitochondrial biogenesis and mitochondrial DNA maintenance of mammalian cells under oxidative stress. Int. J. Biochem. Cell Biol. 37:822–834. 15694841 10.1016/j.biocel.2004.09.010

[36] Chandel, N. S. , and P. T. Schumacker . 1999 Cells depleted of mitochondrial DNA (rho0) yield insight into physiological mechanisms. FEBS Lett. 454:173–176. 10431801 10.1016/s0014-5793(99)00783-8

[37] Lee, H. C. , and Y. H. Wei . 2009 Mitochondrial DNA instability and metabolic shift in human cancers. Int. J. Mol. Sci. 10:674–701. 19333428 10.3390/ijms10020674 PMC2660656

[38] Lee, H. C. , P. H. Yin , J. C. Lin , C. C. Wu , C. Y. Chen , C. W. Wu , et al. 2005 Mitochondrial genome instability and mtDNA depletion in human cancers. Ann. N. Y. Acad. Sci. 1042:109–122. 15965052 10.1196/annals.1338.011

[39] Xu, E. , W. Sun , J. Gu , W. H. Chow , J. A. Ajani , and X. Wu . 2013 Association of mitochondrial DNA copy number in peripheral blood leukocytes with risk of esophageal adenocarcinoma. Carcinogenesis 34:2521–2524. 23803692 10.1093/carcin/bgt230 PMC3810839

[40] Hosgood 3rd, H. D. , C. S. Liu , N. Rothman , S. J. Weinstein , M. R. Bonner , M. Shen , et al. 2010 Mitochondrial DNA copy number and lung cancer risk in a prospective cohort study. Carcinogenesis 31:847–849. 20176654 10.1093/carcin/bgq045 PMC2864414

[41] Du, M. , J. Prescott , M. C. Cornelis , S. E. Hankinson , E. Giovannucci , P. Kraft , et al. 2013 Genetic predisposition to higher body mass index or type 2 diabetes and leukocyte telomere length in the Nurses’ Health Study. PLoS ONE 8:e52240. 23424613 10.1371/journal.pone.0052240 PMC3570546

[42] Argiris, A. , M. V. Karamouzis , D. Raben , and R. L. Ferris . 2008 Head and neck cancer. Lancet 371:1695–1709. 18486742 10.1016/S0140-6736(08)60728-X PMC7720415

[43] Klaunig, J. E. , and L. M. Kamendulis . 2004 The role of oxidative stress in carcinogenesis. Annu. Rev. Pharmacol. Toxicol. 44:239–267. 14744246 10.1146/annurev.pharmtox.44.101802.121851

